# Adjunctive avatar therapy for mentalization-based treatment of borderline personality disorder: a mixed-methods feasibility study

**DOI:** 10.1136/eb-2017-102761

**Published:** 2017-10-22

**Authors:** Caroline J Falconer, Penny Cutting, E Bethan Davies, Chris Hollis, Paul Stallard, Paul Moran

**Affiliations:** 1 NIHR MindTech Healthcare Technology Co-operative, University of Nottingham, Nottingham, UK; 2 Division of Psychiatry and Applied Psychology, School of Medicine, University of Nottingham, Nottingham, UK; 3 Adult Personality Disorder Service, Touchstone Centre, Bethlem Royal Hospital, South London and Maudsley NHS Foundation Trust, London, UK; 4 Department of Health, University of Bath, Bath, UK; 5 School of Social & Community Medicine, Centre for Academic Mental Health, University of Bristol, Bristol, UK

**Keywords:** Personality Disorders, Mental Health

## Abstract

**Background:**

Borderline personality disorder (BPD) is characterised by severe instability in emotions, identity, relationships and impulsive behaviour. One contributing factor to BPD is deficient mentalizing—our ability to understand the mental states of others and ourselves. Psychotherapies can be effective at reducing symptoms of BPD but effects are small. Innovative ways of enhancing existing therapies are therefore essential.

**Objective:**

In a mixed-methods, feasibility and acceptability study, we adjuncted conventional mentalization-based treatment (MBT) for BPD with avatar software (avatar-MBT). We wanted to test whether the enhanced visual narrative afforded by the software would facilitate therapy.

**Methods:**

We used proprietary avatar software in four group MBT sessions. We collected data on uptake (n=15), dropout (n=4) and self-report measures (n=11) of mentalization and mood and conducted qualitative interviews to assess attitudes and beliefs (n=9).

**Findings:**

Thematic analysis revealed five themes on the usefulness of avatar-MBT, including facilitating perspective taking, expression, emotional distancing, the big picture and group participation. The sixth theme suggested avatar-MBT is best placed within a group setting. There was no deterioration in symptoms as monitored by self-report measures.

**Conclusions:**

Qualitative data suggest that avatar-MBT is acceptable to patients with BPD who described it as enhancing conventional MBT and expressed a wish to continue using it. However, controlled trials are required to assess efficacy.

**Clinical implications:**

Results suggest that avatar-MBT may be a viable option to enhance existing BPD treatment. Furthermore, we provide initial evidence that it is feasible to implement a digital adjunct within a group therapy setting.

## Background

Borderline personality disorder (BPD) is a mental disorder characterised by severe instability in emotions, identity, relationships and impulsive behaviour.^1^ The condition occurs globally, with a lifetime community prevalence of up to 6%.^2^ Functional impairment is an enduring feature of the disorder, and people with BPD are at significantly increased risk of suicide, which affects up to 10% of individuals.^3^ Psychotherapies are effective in producing symptomatic remission, although a recent systematic review concluded that effect sizes are relatively small and unstable at follow-up.^4^ A strong case therefore exists for enhancing treatments in order to optimise treatment gains for patients with BPD.

Mentalization-based treatment (MBT) has been shown to reduce suicidal and self-mutilatory acts, depressive symptoms and the number of inpatient days and to improve social and interpersonal functioning for individuals with BPD.^5^ MBT focuses on cultivating mentalization, the process by which we make sense of the thoughts, emotions and behaviours of ourselves and others. Without this capacity, individuals have no coherent sense of self and no stable, predictable and meaningful interaction with others.^6^ This can often result in extreme fluctuations in emotional states, impulsive behaviour and unhealthy attachment to others or a lack of appropriate attachment.

During MBT, the therapist is continually constructing and reconstructing an understanding of the patient’s experience of an interpersonal event, or relationship, to help them comprehend and reflect on their mental state and explore alternative interpretations of the mental states of others.^6^ While cultivating mentalization has been shown to be effective, the process is complex and mentally taxing for both the patient and therapist. Furthermore, not everyone is able to adequately express themselves verbally or in writing.

One potential way of enhancing the MBT process is by adding adjunctive therapy with virtual reality (VR) that allows for an externalised visual representation of the self and others in the form of avatars (virtual bodies). In a small, mixed-methods study, conducted in a male offender population, avatar software was found to be feasible to deliver and may enhance engagement in a wider therapeutic programme.^7^

## Objectives

We set out to conduct a mixed-methods study of the feasibility and acceptability of using an avatar adjunct to MBT (avatar-MBT) in the treatment of individuals with BPD. First of all, we developed the novel avatar adjunct using proprietary VR software. This was then implemented within a personality disorder service with an MBT therapist. We assessed acceptability and feasibility, quantitatively, through self-report measures of mentalization and mood, and recorded uptake and dropout. Self-report measures were primarily used to establish the safety of the intervention by monitoring for symptom deterioration. We also conducted qualitative interviews to assess user experience and attitudes and beliefs towards avatar-MBT. It is possible that avatar-MBT may provide a means of visual narrative that could facilitate the delivery of MBT. Furthermore, we anticipated that the specific avatar perspective taking feature of the software might help participants to mentalize.

## Methods

### Participants

A convenience sample of 15 participants (12 female; age range 20–43 years old; x̅ of 31.2) was recruited through the adult personality disorder service at Bethlem Royal Hospital, South London, and Maudsley NHS Foundation Trust. All participants had a diagnosis of BPD,^1^ as assessed by the Structured Clinical Interview for DSM-IV Axis II ,^8^ and were enrolled in an 18-month outpatient MBT therapy programme that included weekly group-based interventions such as MBT groups, expressive art and writing for mentalizing and recovery-focused practical work. Engagement in therapy was supported through weekly or fortnightly individual MBT sessions. Programme attendance was 2–3 days per week. Ten participants were in the early stages of their therapy, while the remaining five participants were in the later stages, two of which were nearing the end of their therapy. All participants had received, at least, an introductory period of 10 weeks of therapy prior to participating.

Two participants identified as Asian British and one as black British. All other participants were Caucasian. All participants provided written informed consent.

### Software

**Figure 1:**
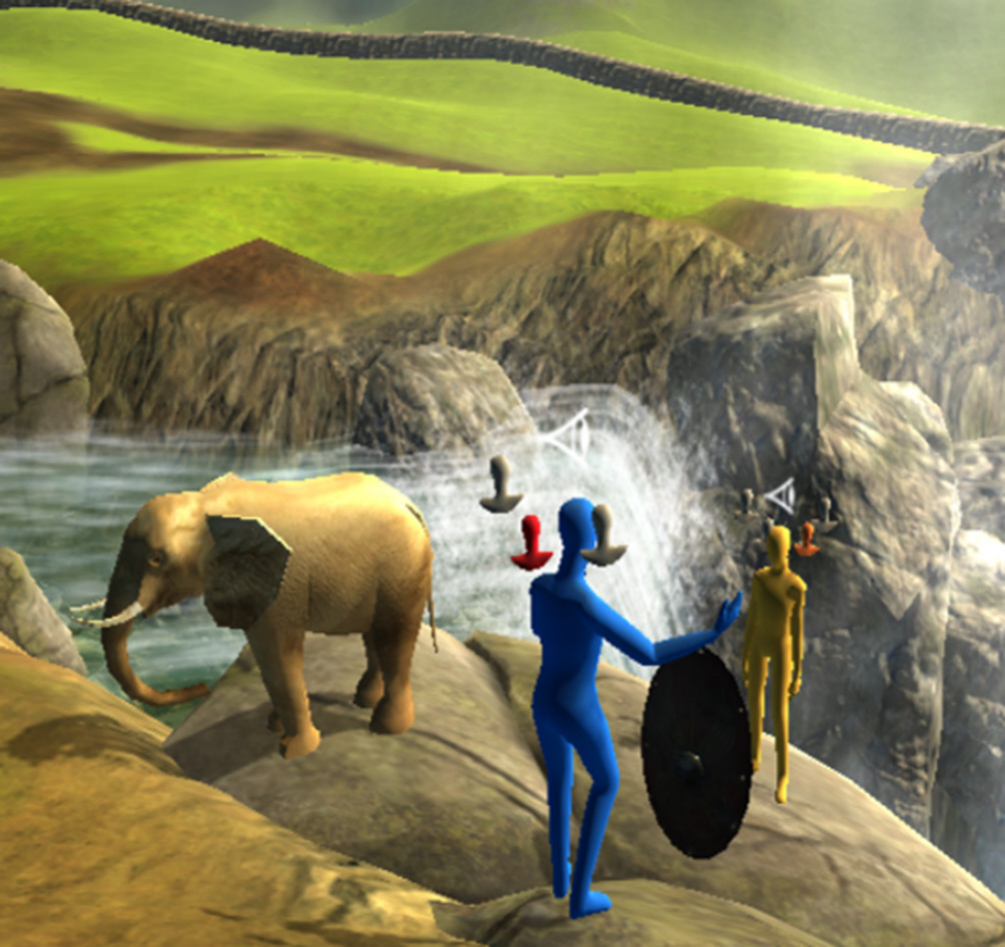
Reconstruction (with permission) of a group VR session depicting the participant’s (yellow avatar, background) relationship to another individual (blue avatar, foreground). The participant represents their precarious relationship at the edge of a cliff next to the waterfall. They also use a shield to represent protection strategies (safety behaviours) in relation to this other individual. The floating heads around each avatar represents different internal monologues that the participant has attributed to themselves and the other individual. Furthermore, the participant has used the elephant object to represent ‘the elephant in the room’ in terms of what is not being discussed between the two individuals.

We used a desktop VR software called ProReal (ProReal, Oxford, UK; www.proreal.world), which is a virtual world and avatar program that helps users create a visual representation of their world (internal and external) and the people in it. Users are able to populate (with support from a therapist) their landscape with virtual people (avatars), which are non-descriptive (without facial features or gender) but can be altered in terms of their size, colour and animation (ie, postures or behaviour). These avatars can also be labelled and given internal monologues and emotions. They can also be controlled through a keyboard, mouse or touchscreen to navigate the environment. Clients have a series of objects available (eg, bridge, clock and treasure chest) to help illustrate and symbolically represent their world. When using the software, users can also change their visual perspective. The ‘free camera’ option allows users to view the virtual environment from any angle, but users can also lock their perspective to an avatar (ie, just behind) or through the eyes of that avatar. A reconstructed scene from one of the group sessions of this study can be seen in figure 1.

### Intervention

**Figure 2:**
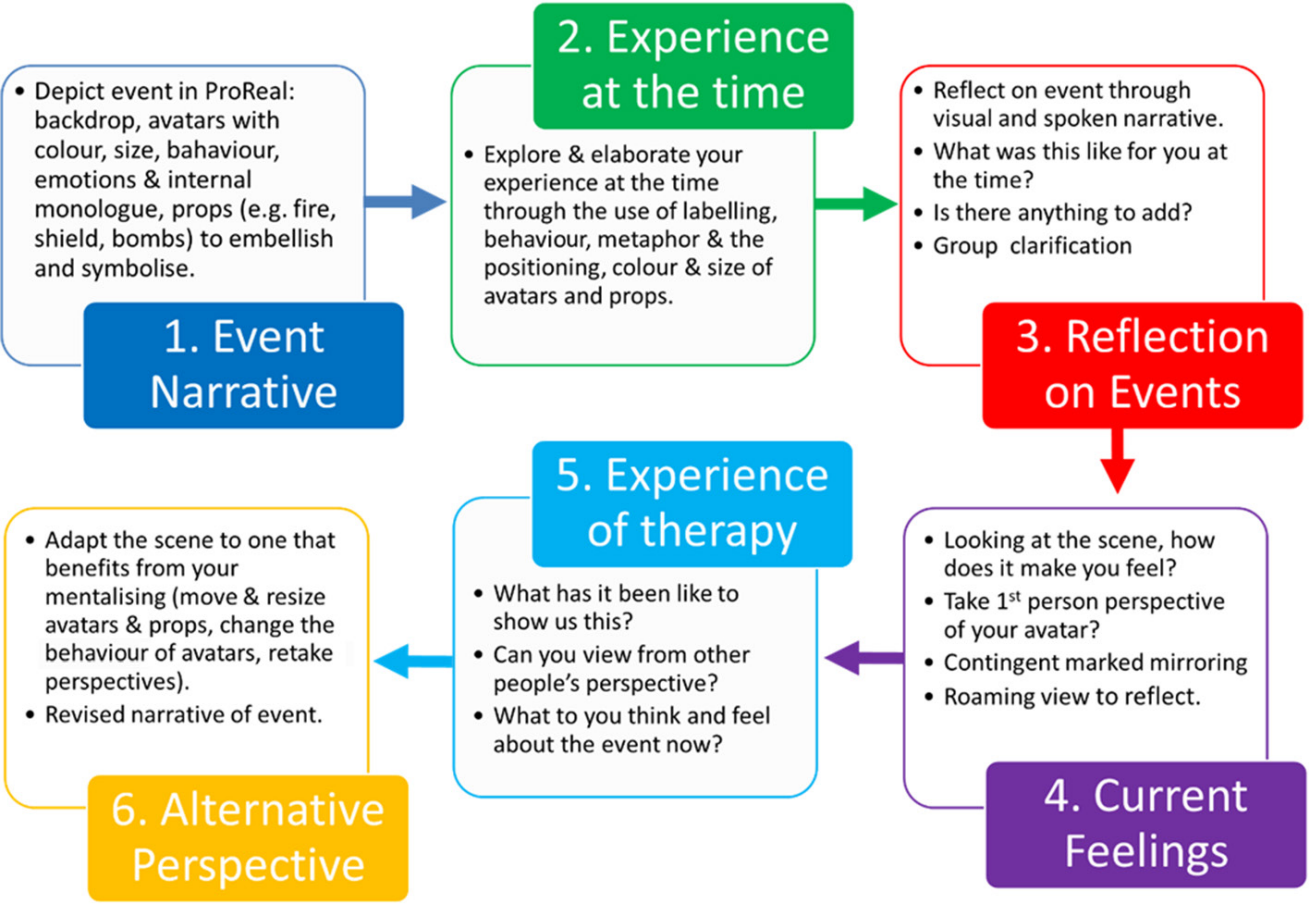
Stages of an avatar-MBT Restoring Mentalizing session (adapted from Bateman and Fonagy 2016). MBT, mentalization-based treatment.

We developed a novel avatar adjunct to MBT (avatar-MBT) based on the mentalizing process as detailed by Bateman and Fonagy^9^ and referred to as a mentalizing functional analysis, whereby the avatar software was used to facilitate ‘Restoring Mentalizing’ group sessions. Avatar-MBT was designed within the research team and with service user involvement. Conventional sessions involve a patient discussing a recent interpersonal event in which they have struggled with or had intense emotional reactions to. The aim of the session is to facilitate joint attention on the mental states of the patient at the time of the event, to help understand the process by which mentalizing became difficult or collapsed. Mentalization is then aided by actively questioning the patient about their experience, linking feelings to actions and moving towards the other group members offering other perspectives about the event. Figure 2 outlines how we integrated key stages of the session with the avatar software. Participants were offered their avatar-MBT sessions in addition to their standard MBT sessions.

### Questionnaires and interview

The 21-item Depression, Anxiety and Stress Scales^10^ has three subscales of seven items relating to depression (eg, ‘I found it difficult to work up the initiative to do things’), anxiety (eg, ‘I felt I was close to panic’) and stress (eg, ‘I found it hard to wind down’). Participants rate the extent to which these items apply to them on a four-point scale (0=‘Did not apply to me at all’; 3=‘Applied to me very much, or most of the time’). Internal consistency for each subscale is α=0.94 for depression, α=0.87 for anxiety and α=0.91 for stress.^11^ Higher scores within the range of 0–21 indicate increased levels of depression, anxiety and stress.

The Mentalization Questionnaire (MZQ)^12^ is a 15-item self-report scale that measures mentalization, either as a whole-scale score or as four subscales: refusing self-reflection (eg, ‘Most of the time I don’t feel like talking about my thoughts and feelings with others’), emotional awareness (eg, ‘Sometimes I only become aware of my feelings in retrospect’), psychic equivalence mode (eg, ‘Often I feel threatened by the idea that someone could criticize or offend me’) and affect regulation (eg, ‘Often I can’t control my feelings’). Participants rate on a 7-point Likert scale the extent to which they agree or disagree with each statement (0=‘Not at all’; 7=‘Very much’). Internal consistency for the total scale is α=0.81, while the subscales were α=0.64, α=0.71, α=0.58, α=0.54, respectively. Higher scores indicate poorer skills on each domain. Scores ranged from 1 to 28 on all subscales except affect regulation, which ranges from 1 to 21.

A brief semistructured interview schedule, consisting of a series of open-ended questions (online supplementary material 1) and prompts, was used to facilitate a semistructured interview with the participant about their experience of using avatar-MBT. The interview guide was developed through discussions within the research team, patient involvement and previous VR research.^13^

SP1 10.1136/eb-2017-102761.supp1 supplementary material

### Procedure

After informed consent, participants completed four group sessions of avatar-MBT at weekly intervals, with a registered practitioner-level MBT therapist. Group sizes ranged from three to six participants. Practical considerations determined the choice of the number of sessions, which were delivered by one therapist in the context of a busy clinical programme. When group attendance was low, avatar-MBT continued in a one-to-one format. Three participants received one one-to-one session, while another participant received two. At each session, participants completed the two questionnaires and sessions lasted approximately 45–60 min. On completion of four sessions, participants took part in the qualitative interview, which was conducted by telephone by *CJF (not involved in therapy delivery)*, recorded on a dictaphone and transcribed verbatim. Interviews ranged between 8 and 24 minutes (x̅=15 minutes).

### Data analysis

For quantitative analysis, in order to assess feasibility and acceptability of avatar-MBT and our study procedures, we recorded the number of participants recruited to and participating in the study, as well as any refusals and dropouts. We also recorded the mean and standard deviation (SD) for symptom scores at each session. We performed individual, repeated measures analysis of variance (ANOVA) on each of the self-report measures completed across the four avatar-MBT sessions, and analysis was completed using SPSS V.23.

Qualitative analysis was undertaken through the six-step approach to thematic analysis,^14^ using nVivo (V.11, QSR International) to support the iterative process. Thematic analysis involves identifying patterns and trends in qualitative data and is done through familiarising oneself with the data, generating and assigning codes, sorting codes into meaningful ‘themes’ and reviewing and identifying themes.^14^ An inductive, ‘bottom-up’ approach was used to code data with descriptive labels and merge codes together to identify emergent themes. All transcripts were read individually by CJF and EBD, with each researcher annotating their own initial descriptive codes. They then discussed their codes to identify and collapse similar codes into initial themes and developed a descriptor for each theme, and then recoded the data with the initial themes. After this was done, both researchers contrasted their assigned themes, identified agreements and discussed and resolved differences in assigned themes.

## Findings

Fifteen participants were recruited from a total pool of 28 patients (43% response). Two patients from this pool actively refused participation, and the remaining 11 patients did not express interest after the study was introduced. Of the 15 participants recruited to the study, four dropped out of treatment altogether (27%). Of the 11 remaining participants, all competed four avatar-MBT sessions and nine (eight females) made themselves available for qualitative interviews.

Repeated measures ANOVA revealed no significant changes for any self-report measures across the four sessions (smallest p=0.12). One participant had only partial data from the MZQ for the first session. Table 1 summarises these results.

**Table 1:** Averages (SD) of self-report measures across avatar-MBT sessions

	Avatar-MBT session			
	1	2	3	4
DASS-21 depression	13.7(4.0)	12.7 (4.6)	14.2 (5.1)	13.4 (4.8)
DASS-21 anxiety	12.3 (4.5)	11.6 (5.4)	11.6 (5.1	11.5 (6.8)
DASS-21 stress	13.9 (4.3)	13.2 (5.0)	12.5 (4.1)	14 (5.2)
MZQ total	81.3 (8.4)	75.9 (16.8)	81.8 (11.7)	74.5 (17.5)
MZQ refusing reflection	21.2 (3.1)	19.5 (5.2)	21.9 (4.0)	18.6 (6.2)
MZQ emotional awareness	22.2 (3.4)	19.4 (6.6)	21.3 (3.7)	19.5 (4.6)
MZQ psychic equivalence	22.4 (3.3)	22.1 (4.8)	22.9 (4.7)	20.5 (5.5)
MZQ affect regulation	15.5 (2.3)	14.9 (3.3)	15.7 (2.8)	15.9 (3.7)

DASS-21, Depression Anxiety and Stress Scales; MBT, mentalization-based treatment; MZQ, Mentalization Questionnaire.

The qualitative analysis resulted in six key themes, one of which related to the positioning of avatar-MBT within a specific therapy context, while the others focused on how participants found avatar-MBT helpful. There was an overwhelmingly positive response to the avatar-MBT, and all participants expressed a wish to continue using the software in therapy. Avatar-MBT was often compared with standard MBT sessions, with participants reporting that avatar-MBT had more ‘*impact*’ or was more ‘*powerful*’ than standard MBT. When probed further about this, the following themes emerged.

###  Visualisation helps me to express and understand myself

The visual nature of the software and its interactive functions provided participants (n=9) with a means of expression and, through this, a developed understanding of themselves. In particular, through the process of visual narrative, participants could identify and label thoughts, emotions and behaviours and recognise the relationships between them:

"I thought it was good to visualise it and make connections through, sort of like, what you were seeing, or what you were perceiving, in the posture and what you felt at the time. And how you thought other people felt at the time and sort of relating that to how the posture was." (Female, 31 years old)

"Yeah the floating heads *[internal monologues]*. I liked, you know, being able to re-size them and give them different colours and just kind of see how big they are in perspective to everything else….I think it was just helpful to see, you know, how important a certain thought or feeling was in certain situations, for each of the avatars." (Female, 20 years old)

Participants also found that they could express themselves through the abstract and symbolic nature of the avatar software:

"I just found colours I can connect to emotions and sizes connect to emotions and feelings as well." (Female, 33 years old)

###  Visual narrative helps me to keep track and participate

Standard MBT sessions require a level of detail to microslice situations in order to identify where mentalizing has collapsed, and this can be demanding for therapists and patients. In contrast, the visual cues of avatar-MBT proved easier to concentrate on (n=9):

"When it came to other people, watching them do it, I always found it really, really helpful because I often have trouble concentrating and, like, following what’s happening, if someone’s just talking. And so like seeing it physically and going through it bit by bit, it was really helpful. I could give more feedback than usual." (Female, 27 years old)

Furthermore, the presence of visual information and the level of detail captured seemed to facilitate group discussion (n=5):

"I think that it really got everyone in the group talking as well. And it had more visual so everyone could see… When it’s on the screen it’s easier to be able to see it and remember it quickly." (Female, 23 years old)

### Avatars help me take and understand another’s perspective

Participants reported that the use of avatars and, more specifically, the use of the avatar perspective tool facilitated their ability to take and understand the perspectives of others (n=5):

"It’s literally right there in front of you on the screen. You could literally see what was going on for other people. You were able to kind of mentalize about it by, you know, writing down what they might have been thinking or they might have been feeling in the situation and it was just, it felt easier to just notice that." (Female, 20 years old)

### Allowing me to see the big picture

While the software allowed participants to set out and discuss a situation ‘thoroughly’ and ‘in more detail’, the use of the software also seemed to enhance the participant’s ability to gain a clearer sense of the ‘big picture’ or the situation as a whole (n=7).

### Giving me distance to think clearly

Participants explained that the avatar-MBT allowed them to externalise their emotions and thoughts (n=7):

"To take all of those thoughts and feeling out of my head and my body and put it in front of me, as a separate thing is kind of revolutionary for me… [when] my mood is governed by something that happened earlier I think all my thoughts and it’s hard to separate those. When you put them separate from you, out there, you kind of look at it from a different aspect and [are] not governed by those things." (Male, 34 years old)

### Group therapy is best, but one-to-one sessions have value too

When asked about the therapy context in which avatar-MBT should be used (eg, group, one-to-one and self-guided), participants (n=9) said that the software was best placed as a part of group therapy. This was because of the need for others to help with the mentalizing process:

"I think using it in a group was probably more helpful because other people could have given their perspective on what the different avatars were thinking or feeling. Whereas when you’re on your own you have to think about it all yourself and you might miss something." (Female, 20 years old)

However, participants did see the advantages of having one-to-one sessions if there was a more personal topic in need of discussion or one that required a further, more in-depth exploration. There was some interest expressed in undertaking a self-guided session but also reservations about having to mentalize independently and the skills required for this.

### Suggested improvements

Participants were asked whether they found the software unhelpful in any way, but no participant could think of examples. Four participants reported software glitches, but this did not impact their overall experience of avatar-MBT. When asked about improvements to the software or the delivery of avatar-MBT, five participants said that they would like to see a variety of environments beyond the current rural version of the software. Some felt that they could better relate to an urban or more social setting.

## Discussion

The results from this study show that avatar-MBT is a promising enhancement to therapy for BPD. Furthermore, our qualitative findings suggest that using the software may enhance the therapeutic efficacy of standard MBT. All participants wanted to continue with the use of avatar-MBT as part of their therapy and, importantly, no participants deteriorated during the course of the study. We did not detect any improvements in the self-report measures of mentalization or mood, but our sample size was small and assessments took place over a short timescale within the context of an 18-month programme of treatment.

Our qualitative data suggest avatar-MBT makes abstract discussion more concrete. First of all, we suggested that the use of the perspective-taking function could facilitate mentalization. Participant responses indicated that this was indeed the case. Participants stated that through this function, they could ‘*literally*’ see a situation or how they had behaved from another person’s perspective. This visual change in perspective appears to have facilitated the participants’ ability to think about the mental states of others. While one participant acknowledged that they may never truly know what another person thought at a specific moment in time, they said that a visual shift in perspective opened up the prospect of a different interpretation of an event. Exploring potential perspectives (visual or cognitive) of others is an inherent part of MBT and participants told us that the avatar software made this process easier.

Second, participants reported that the software provided them with a holistic view of a situation and the people involved. The software therefore appeared to facilitate the enhancement of reflective functioning. Participants reported ‘stepping back’ to take in the ‘big picture’ and thus gain fresh insights into the situations they were encountering during daily life.

Third, while participants highlighted the positive, powerful nature of the software, paradoxically they also suggested that the software afforded a level of distance between themselves and the emotional distress that they often felt when describing a significant event. BPD is characterised by intense emotional reactions and high levels of arousal. While this is a consequence of difficulties in mentalization, intense emotions and high arousal also inhibit our ability to mentalize.^15^ Therapists try to lower this arousal during sessions to facilitate mentalization, but this can be challenging, and our data suggest that the software may help participants to better manage arousal during the course of therapy. Visual externalisation and an enhanced sense of control may be important elements in the mechanism of effect and is a similar process adopted in avatar therapy for auditory hallucinations.^16 17^

Fourth, the visual nature of avatar-MBT and the features it offers (eg, labelling, internal monologues and symbolic representation through colour, size and body posture) allowed participants to express themselves and explain situations or relationships. This contributed to an increased understanding of their own thoughts, emotions and behaviours and the relationships between them. This was often discussed in relation to standard MBT whereby they had to conduct this verbally, which was a struggle for many.

Finally, participants reported that the visual and interactive qualities of avatar-MBT increased their ability to concentrate and participate in sessions. This is particularly important for BPD group therapies, where it is important to encourage inquisitiveness, questioning and collaboration. The software also appeared to have improved the attentional focus of the group members. This could have implications for developing effortful control skills to combat poor impulse control seen in people with BPD.^18^

This was a small feasibility and acceptability study, and our results have to be interpreted in light of this. Furthermore, the avatar-MBT was conducted as part of a wider therapeutic programme, and we cannot rule out the contribution of ongoing therapies to our findings. The study also did not address technical and information governance issues relating to implementation of avatar-MBT within routine mental health service settings or feedback from therapists. Our participants were also almost all female, and we cannot generalise our results to male participants. Finally, the study was conducted within one service with one therapist, and our results may not be generalisable to other teams.

## Clinical implication

Avatar-MBT is a promising approach for the treatment of BPD and appears acceptable to patients. While our study was conducted in one service, with a motivated team, we have also demonstrated that it is feasible to integrate digital technology into a traditional psychotherapy setting and at a group level. Our results offer encouragement to both patients and therapists and suggest that the potential clinical, service efficiency and health economic benefits of avatar-MBT should be tested in larger controlled studies with different therapists and services. An important question could be whether avatar-MBT might accelerate or shorten the duration of MBT, thereby enhancing adherence and reducing costs. The majority of our participants were at the beginning of therapy, and our results might suggest avatar-MBT is best placed within the early stages of therapy, which may also maximise any cost-effectiveness benefits.
