# Supplementary material for: Adjunctive avatar therapy for mentalization-based treatment of borderline personality disorder: a mixed-methods feasibility study
Source: Evid Based Ment Health. 2017 Oct 22;20(4):123–7. doi: 10.1136/eb-2017-102761 (PMC5750410; doi:10.1136/eb-2017-102761)
Supplement: supplementary material [file ebmental-20-123-DC1-inline-supplementary-material-1.pdf]

The University of Nottingham
UNITED KINGDOM · CHINA · MALAYSIA

Supplementary Material 1

Avatar-MBT: Interview Schedule

v1 18.12.15

1. **How was your experience of using ProReal?**

    a) Was it easy to use?
    b) Was the software engaging/inviting/interesting?
    c) Did the software do what you wanted it to do?
    d) Were there any features that you liked at lot or used a lot?
    e) Were there any features that you did not like or did not use a lot?

2. **Have you found your experience with ProReal (un)helpful*?***

    a) Was there any instance when ProReal was helpful?
    b) What changes do you think have happened because of using ProReal?
    c) Have you learned anything after using ProReal during therapy? What?
    d) Were there any specific features that were useful? What were they?
    e) Was there any instance where ProReal was unhelpful or got in the way?

3. **What did you think about the number of ProReal sessions?**

    a) Was 4 sessions enough or would you have preferred less or more sessions? Why?
    b) Do you have a preference for using ProReal in the one-to-one or group therapy sessions? Why? What about self-guided?

4. **Do you have any questions or would you like to add anything?**
